# Molecular Survey of Cell Source Usage during Subtotal Hepatectomy-Induced Liver Regeneration in Rats

**DOI:** 10.1371/journal.pone.0162613

**Published:** 2016-09-15

**Authors:** Andrey Elchaninov, Timur Fatkhudinov, Natalia Usman, Evgeniya Kananykhina, Irina Arutyunyan, Andrey Makarov, Galina Bolshakova, Dmitry Goldshtein, Gennady Sukhikh

**Affiliations:** 1 Research Center for Obstetrics, Gynecology and Perinatology of Ministry of Healthcare of the Russian Federation, 4 Oparina Street, Moscow 117997, Russia; 2 Scientific Research Institute of Human Morphology, 3 Tsurupa Street, Moscow 117418, Russia; 3 Pirogov Russian National Research Medical University, Ministry of Healthcare of the Russian Federation, 1 Ostrovitianov Street, Moscow 117997, Russia; 4 Research Centre of Medical Genetics, 1 Moskvorechie Street, Moscow 115478, Russia; University of Navarra School of Medicine and Center for Applied Medical Research (CIMA), SPAIN

## Abstract

Proliferation of hepatocytes is known to be the main process in the hepatectomy-induced liver regrowth; however, in cases of extensive loss it may be insufficient for complete recovery unless supported by some additional sources e.g. mobilization of undifferentiated progenitors. The study was conducted on rat model of 80% subtotal hepatectomy; the objective was to evaluate contributions of hepatocytes and resident progenitor cells to the hepatic tissue recovery via monitoring specific mRNA and/or protein expression levels for a panel of genes implicated in growth, cell differentiation, angiogenesis, and inflammation. Some of the genes showed distinctive temporal expression patterns, which were loosely associated with two waves of hepatocyte proliferation observed at 2 and 7 days after the surgery. Focusing on genes implicated in regulation of the progenitor cell activity, we came across slight increases in expression levels for Sox9 and two genes encoding tumor necrosis factor-like cytokine TWEAK (Tnfsf12) and its receptor Fn14 (Tnfrsf12a). At the same time, no increase in numbers of cytokeratin 19-positive (CK19^+^) cells was observed in periportal areas, and no CK19^+^ cells were found in hepatic plates. Since CK19 is thought to be a specific marker of both cholangiocytes and the hepatic progenitor cells, the data indicate a lack of activation of the resident progenitor cells during recovery of hepatic tissue after 80% subtotal hepatectomy. Thus, proliferation of hepatocytes invariably makes the major contribution to the hepatic tissue recovery, although in the cases of subtotal loss this contribution is distinctively modulated. In particular, induction of Sox9 and TWEAK/Fn14 regulatory pathways, conventionally attributed to progenitor cell activation, may incidentally stimulate mitotic activity of hepatocytes.

## Introduction

Regeneration of mammalian liver has inspired a huge amount of research. Most of the studies have been done on laboratory rodents using the partial hepatectomy model, that is, by excision of median and left lobes, which constitute about 70% of the organ volume [1]. The procedure is currently standardized and commercially available [2], whereas variations in the removed volume may seem to be less revealing, as long as a much smaller number of studies are dealing with them [3,4].

Nonetheless, resections of an extremely large volume (80–90%) of the organ represent a special interest due to their clinical relevance. The management of small size hepatic remnant still remains a challenge [5], and the signs of acute hepatic failure frequently develop during transplantation of liver tissue when the graft is too small to maintain the homeostasis. A similar response at the systemic level is typical for the patients whose livers have been subjected to extensive resection leaving an extremely small part of the organ, in order to withhold a tumor from spreading [5,6].

A number of genes with differential expression have been linked to certain critical points of liver regeneration; the list includes cytokines (Il1b, Il6, Il10), growth factors (Hgf, Tgfb, Fgf2, Tnfa) [7,8,9], and other regulatory molecules. Notably, descriptions of expression pattern for a particular molecule may differ in details. For example, some of the authors have found that expression of Il1b, Il6, Il10, Hgf, Tgfb, and Fgf2 genes stays elevated for 2–3 days after the subtotal resection [10,11,12], whereas others describe it as series of short outbursts or as a single spike [6].

According to the classical view, the liver subjected to partial hepatectomy regenerates by proliferation of hepatocytes and, to a lesser extent, cholangiocytes, but not the immature and undifferentiated cells [9]. This view is currently questioned [13], and it would be more correct to say that contribution of resident progenitor cells to liver regeneration depends on nature and severity of the inducing damage, because any violation of proliferation capacity of hepatocytes or cholangiocytes may create a niche for participation of hepatic progenitor cells (HPC) [13]. Although the role of HPC in liver regeneration in cases of extensive loss has not been fully elucidated, it is known that proliferation of hepatocytes in such cases is temporarily blocked [14], and this delay may promote participation of the resident progenitors. Molecular mechanisms regulating activation and differentiation of HPC are being extensively studied, and the TWEAK-signaling pathway is currently positioned as their principal component. TWEAK, the TNF ligand family member known for its general pro-angiogenic capacities, acts through its receptor Fn14 to activate NF-κB signaling [15]. TWEAK is a potent inducer of HPC proliferation [16,17,18]; in particular, it has a selective mitogenic effect for the liver progenitor ("oval") cells in rodents, while an increased Fn14 expression in chronic hepatitis C and related diseases suggests a similar role for the TWEAK/Fn14 pathway in humans [19].

The objective of current study comprised an updated characterization of liver regeneration from small residue in terms of gene expression along with assessment of the contributions from hepatocytes and HPC.

## Materials and Methods

### Experimental model

The borderline condition produced by subtotal 80% hepatectomy in a rat is described as an acute liver failure. The condition is resolved in the course of 48 h by either spontaneous death of the animal or switch to the rapid recovery (S1 Fig), and no means for apriori distinction between the survivors and non-survivors have been reported. Despite the concerns about the inevitability of uneuthanized spontaneous death of certain part of operated animals, but taking into account the well-established character, consistency, and continuous history of the model, the Ethical Review Board at the Scientific Research Institute of Human Morphology have decided to approve the study (Protocol No. 5, March 12, 2013). Outbred male Sprague-Dawley rats, body weight 300–400 g, were obtained from Institute for Bioorganic Chemistry branch animal facilities (Pushchino, Moscow region, Russia). All experimental work involving animals was carried out according to the standards of laboratory practice (National Guidelines No. 267 by Ministry of Healthcare of the Russian Federation, June 1, 2003), and all efforts were made to minimize suffering.

Although any experimental study of liver regeneration is conventionally fitted to the long-established general scheme, the design inevitably requires specifications related to particular tasks of the study, with basic challenges represented by the reasonable choice of the control (either quite intact, or sham operated) and the proper consideration of confounding factors that include the surgically induced stress response and systemic manifestations of the acute liver failure, but also the correct interpretation of the variation between individual animals within the groups and the means of accounting for such variation in order to increase reliability of statistical handling of the data.

The animals were operated as described elsewhere [2], under general anesthesia with diethyl ether (Medhimprom, Moscow region, Russia; 0.08 ml per liter of chamber volume [20]), between 9 am and 11 am. Diethyl ether is commonly used as an anesthetic in modeling liver regeneration via hepatectomy [4,12]. In our setting, it provided the least negative effect on animal survival as compared with its alternatives, Zoletil ®100 and isoflurane, probably due to its high pulmonary clearance rates (estimated 90% of the absorbed diethyl ether is exhaled unchanged) in combination with the minimal contribution of hepatic metabolism to the rates of its excretion.

To access the liver, ventral and midline longitudinal incisions were made to the skin and abdominal wall at the level of the organ; padded retractors were used to increase surgical visibility. The liver was externalized, and the vessels of the median, lateral, and superior right lobes of the liver were permanently ligated; subsequently, the median, left and superior right lobes were excised (approximately 80% organ removal) and fixed as control samples for gene expression analysis (see below). The remaining lobes, i.e. the inferior right and caudate, kept moist throughout the procedure with sterile saline, were returned to their initial position within the abdominal cavity, and the field was flushed with sterile saline once again. The ventral and dorsal incisions were closed with suture and treated with 0.05% chlorhexidine bigluconate followed by an alcohol swabbing.

The operated animals, two per cage, were housed for recovery in a temperature-regulated room with a 12:12 h light-dark cycle and unlimited access to food and water. The animal health was inspected 4 times a day during the first 48 h after the surgery, and subsequently 2 times a day until the sacrifice. Meloxicam (1.0 mg/kg/day) was repeatedly injected into the neck fat pad of the animals as postoperative analgesia for two days after the surgery; additionally, gentamicin (3.0 mg/kg/day) was injected subcutaneously as an antibiotic on the first day after the surgery.

The animals were drawn from the experiment in CO_2_-chamber at 3 h, 6 h, 12 h, 24 h, 30 h, 48 h, 72 h, 5 days, 7 days, or 10 days after the surgery (5–6 animals for each term); the entire mass of regenerating liver tissue was promptly dissected and weighed prior to analysis.

All the liver tissue removed in the course of the surgery was carefully collected and conserved for further examinations. In particular, the quantitative data on gene expression for the tissue of left lobe obtained by resection at the day of the surgery was directly compared with the corresponding data for the right lobe of the same animal, harvested 3 hours to 10 days after the surgery; this algorithm is recommended to minimize variation inherent in comparing pre- and postoperative livers of different animals and to increase the power of statistical approaches for detecting differences in gene expression [21]. Alternatively, hepatic tissue from non-operated or sham-operated animals served as additional control in the assessment of the liver mass recovery and hepatocyte proliferation, functional tests, immunostaining, and western-blot analysis. The sham operation procedure exactly reproduced all steps of the surgery, with the exception that the liver lobes were only briefly externalized and then returned into their original position. The non-operated control group included intact male rats (n = 10) matching all the experimental group parameters.

### Functional tests

Recovery of liver function was assessed by serum albumin concentration and alanine aminotransferase (ALT) tests. The albumin concentration was determined by Albumin DiaS® photometric test with bromocresol green (DIAKON-DS, Pushchino, Russia), and the ALT activity was measured by GPT Dias® photometric test (DIAKON-DS), according to the manufacturer protocols.

### Light microscopy and cell counts

The tissues were fixed in 10% neutral-buffered formalin (BioVitrum, St. Petersburg, Russia), subsequently dehydrated, embedded in paraffin, and sectioned using a rotary microtome; the 5–7 μm sections were stained with hematoxylin and eosin (BioVitrum St. Petersburg, Russia).

Hepatocyte proliferation was assessed by calculation of mitotic index expressed in parts per thousand (‰). Mitotic figures were counted manually per 6×10^3^ cells for each animal.

### Immunostaining

Primary antibodies to Sox9 or cytokeratin 19 (CK19; Abcam, Cambridge, UK) were applied in 1:100 dilution following pretreatment of the slides with sodium citrate buffer (10 mM sodium citrate, 0.05% Tween 20, pH 6.0, pre-heated to boiling in accordance with the heat-induced epitope retrieval protocol recommended by the manufacturer). Signal development was carried out by immunoperoxidase procedure; the slides were counterstained with hematoxylin.

The Sox9^+^ и CK19^+^ cells were manually counted on immunostained slides, and the corresponding indexes were calculated as proportions of positive cells (%).

### Western-blot analysis

The content of Sox9 and cytokeratin 19 (CK19) proteins in liver tissues was quantified by Western-blot analysis using the equipment, consumables, and protocols by Bio-Rad Laboratories, Inc. (Hercules, CA, USA). Isolation of proteins from the liver tissue was carried out using MicroRotofor™ Cell Lysis Kit, the total protein content was measured by Bradford assay using Quick Start™ Bovine γ-Globulin Standard, and the Trans-Blot® Turbo™ RTA Mini LF PVDF Transfer Kit was used for the transfer of resolved proteins from the gel to polyvinylidene fluoride blotting membrane. Other Bio-Rad products, used in this section, included the Immun-Star Goat Anti-Rabbit (GAR)-HRP Conjugate as the secondary antibodies, Clarity™ Western ECL with ChemiDoc™ system for the signal development, and the Image Lab™ software for the data analysis. Primary antibodies to Sox9, CK19, or β-tubulin (all by Abcam) were applied in 1:100 dilutions, as recommended by the manufacturer.

### Polymerase chain reaction (PCR)

Expression of molecular markers was analyzed by real-time quantitative reverse transcription PCR. Slices of tissue from the liver, the lung, or the kidney, each about 30 mm^3^ volume, were submerged in RNAlater RNA Stabilization Reagent (QIAGEN, Hilden, Germany) immediately after harvesting, incubated overnight at 4°C, and stored at -80° until use. The tissue from the excised lobes of the liver was used as a reference for analysis of gene expression in regenerating livers; fragments of lung and kidney from intact animals were used as reference samples for analysis of gene expression in corresponding organs of the operated animals.

Total RNA was isolated from the tissue samples using RNeasy Plus Mini Kit (QIAGEN) according to the manufacturer protocol. Estimated purified RNA concentration in the eluate was 0.1 g/l; quality of the material was controlled by electrophoresis.

To remove traces of genomic DNA, the RNA was treated with an aqueous solution of DNase I, RNase-free (Thermo Scientific, Waltham, MA, USA; 1 U per μg of the RNA, in the presence of Mg^2+^, followed by heat inactivation of DNAse according to the manufacturer protocol); the efficacy was confirmed by PCR with primers specific to nontranscribed genomic regions. Reverse transcription of the total RNA to randomly primed single-stranded cDNA was done using MMLV RT Kit (Evrogen CJSC, Moscow, Russia); the synthesis was carried out at 39°C for 1 hour. After heat inactivation of the enzyme, the reaction mixture was diluted with 2 volumes of TE buffer (10mM Tris pH 8.0, 0.1mM EDTA) for further use and storage; final dilution of the mixture in PCR constituted 1:250.

Polymerase chain reactions were set in duplicates on the basis of qPCRmix-HS SYBR (Evrogen CJSC) with oligonucleotide primers (custom made by SYNTOL, Moscow, Russia) in 0.2–0.4 μM final concentrations. Structures of the oligonucleotides with symbols and descriptions of the corresponding targets are given in Table 1. Amplification with detection and digital analysis of fluorescence in real-time was carried out on DT-96 Real-Time PCR Cycler (DNA-Technology JSC, Moscow, Russia) in standard mode of 95°C for 5 min followed by (95°C 15 s, 62° for 10 s +reading, 72° for 20 s) × 45.

**Table 1:** PCR primer structures and targets definition.

target symbol	direction	sequence (5′ to 3′)	NCBI Ref Seq	target definition
*ang*	forward	GCGAATGGAAGCCCTTACG	NM_001012359.1	*Rattus norvegicus* angiogenin, ribonuclease A family, member 2 (Ang2), mRNA
	reverse	GCCTGCTGAGTAGAGACTGAT		
*fgf2*	forward	CCAAGCGGCTCTACTGCAA	NM_019305.2	*R*. *norvegicus* fibroblast growth factor 2 (Fgf2), mRNA
	reverse	AGCCGTCCATCTTCCTTCATAG		
*fn14*	forward	GGA CTG CGC TTC TTG TCC A	NM_181086.3	*R*. *norvegicus* tumor necrosis factor receptor superfamily, member 12a (Tnfrsf12a), mRNA
	reverse	GCA GCC TTC TCC ACC AGT CT		
*hgf*	forward	GGCCATGGTGCTACACTCTT	NM_017017.2	*R*. *norvegicus* hepatocyte growth factor (Hgf), mRNA
	reverse	TTGTGGGGGTACTGCGAATC		
*il10*	forward	GCC CAG AAA TCA AGG AGC AT	NM_012854.2	*R*. *norvegicus* interleukin 10 (Il10), mRNA
	reverse	TGA GTG TCA CGT AGG CTT CTA		
*il1b*	forward	CTG TCT GAC CCA TGT GAG CT	NM_031512.2	*R*. *norvegicus* interleukin 1 beta (Il1b), mRNA
	reverse	ACT CCA CTT TGG TCT TGA CTT		
*il6*	forward	TAC ATA TGT TCT CAG GGA GAT	NM_012589.2	*R*. *norvegicus* interleukin 6 (Il6), mRNA
	reverse	GGT AGA AAC GGA ACT CCA G		
*iNOs*	forward	CGC TGG TTT GAA ACT TCT CAG	NM_012611.3	*R*. *norvegicus* nitric oxide synthase 2, inducible (Nos2), mRNA
	reverse	GGC AAG CCA TGT CTG TGA C		
*mmp9*	forward	ATGGTTTCTGCCCCAGTGAG	NM_031055.1	*R*. *norvegicus* matrix metallopeptidase 9 (Mmp9), mRNA
	reverse	CACCAGCGATAACCATCCGA		
*sdfa*	forward	TGCCGATTCTTTGAGAGCCA	NM_022177.3	*R*. *norvegicus* chemokine (C-X-C motif) ligand 12 (Cxcl12), transcript variant 1, mRNA
	reverse	TCCTTTGGGCTGTTGTGCTT		
*sox9*	forward	AGA GGC CAC CGA ACA GAC T	XM_003750950.3	*R*. *norvegicus* PREDICTED: SRY (sex determining region Y)-box 9 (Sox9), mRNA
	reverse	TGC TCA GCT CAC CGA TGT C		
*tgfb*	forward	CCGCAACAACGCAATCTATG	NM_021578.2	*R*. *norvegicus* transforming growth factor, beta 1 (Tgfb1), mRNA
	reverse	AGCCCTGTATTCCGTCTCCTT		
*tnfa*	forward	CCA CCA CGC TCT TCT GTC TA	NM_012675.3	*R*. *norvegicus* tumor necrosis factor (Tnf), mRNA
	reverse	GCT ACG GGC TTG TCA CTC G		
*tweak*	forward	GAT GGA GCA CAG GCA GGT G	NM_001001513.2	*R*. *norvegicus* tumor necrosis factor ligand superfamily member 12 (Tnfsf12), mRNA
	reverse	TGG CTG AGA ATT CTT CCA G		
vegf*	forward	GCAGCGACAAGGCAGACTAT	NM_031836.2	*R*. *norvegicus* vascular endothelial growth factor A (Vegfa), transcript variant 1, mRNA
	reverse	GAGGGAGTGAAGGAGCAACC		
*actb*	forward	GAGATTACTGCCCTGGCTCC	NM_031144.3	*R*. *norvegicus* actin, beta (Actb), mRNA
	reverse	GCTCAGTAACAGTCCGCCTA		
*b2m*	forward	CTC GCT CGG TGA CCG TGA T	NM_012512.2	*R*. *norvegicus* beta-2 microglobulin (B2m), mRNA
	reverse	GGA CAG ATC TGA CAT CTC GA		
*gapdh*	forward	GCGAGATCCCGCTAACATCA	NM_017008.4	*R*. *norvegicus* glyceraldehyde-3-phosphate dehydrogenase (Gapdh), mRNA
	reverse	CCCTTCCACGATGCCAAAGT		

* The *vegf*-specific primers also match NM_001110333.1, NM_001110334.1 (*R*. *norvegicus* Vegfa transcript variants 2 and 3).

Characteristic values (Cp) were automatically generated by nonlinear regression analysis, and the relative expression values were calculated by approach originally introduced by Pfaffl [22] with modifications by Vandesompele *et al*. [23] using *actb*, *b2m*, and *gapdh* (see Table 1) as reference targets.

### Statistics

The data were analyzed using SigmaStat 3.5 (Systat Software Inc., Chicago, IL, USA). Sample proportions were compared by 2-sample z-test; relative gene expression values were compared by the Mann-Whitney U test; more-than-two-groups comparisons were done using ANOVA on ranks; *p*<0.05 for the differences were considered statistically significant.

## Results

### Animal survival

**Fig 1:**
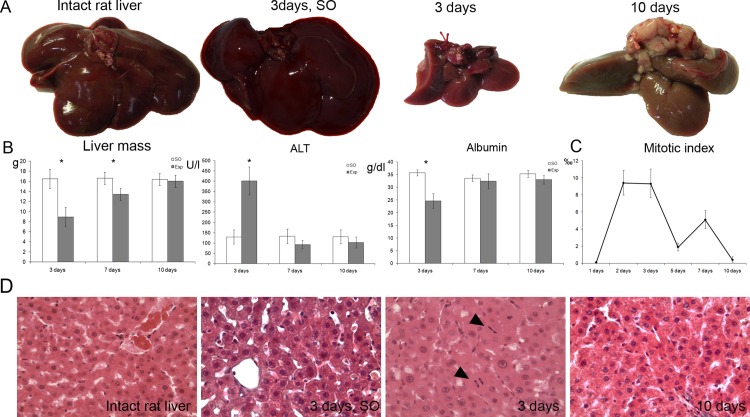
Recovery rates of liver after 80% subtotal hepatectomy in rats. (**A**) Overall view of gross specimens in the intact state and at different days after the surgery; the plot shows recovery of the initial organ mass as measured at different days after the surgery and compared to the sham operated controls. (**B**) Serum concentrations of ALT and albumin in hepatectomized and sham operated rats. (**C**) Mitotic activity of hepatocytes in residual livers after 80% subtotal hepatectomy plotted against time elapsed after the surgery. No mitotic figures are observed in hepatocytes of the sham operated livers; the mitoses are induced by the surgery. Exp–operated rats, SO—sham operated rats. The data are represented as mean values ± SEM with asterisks indicating statistical significance of differences (as compared to the control; *p*<0.05).

Some of the animals died over the first two days after the surgery, giving total mortality of approx. 50%; this rate is consistent with the literature [10]. Apparently, it was associated with the acute hepatocellular dysfunction indicated by a decrease in serum albumin concentration parallelled by an increase in serum ALT activity, which sustained in the hepatectomized rats for 3 days following the intervention (Fig 1).

### Recovery of liver mass and function

Residual livers reached the initial volume of the organ by 10 days after the surgery (Fig 1A). The albumin concentration and ALT activity in serum returned to their original values by the same day, indicating functional recovery (Fig 1B). On this basis, the ten-day compensatory growth was considered complete.

### Mitotic activity of hepatocytes

Although no mitotically active hepatocytes were observed in the regenerating livers for the first 24 h after the surgery, this delay was followed by a sharp rise in their numbers. On the whole, the mitotic index of hepatocytes peaked twice, at 48 to 72 h and at 7 days after the surgery (Fig 1C).

### CK19 and Sox9 protein expression

**Fig 2:**
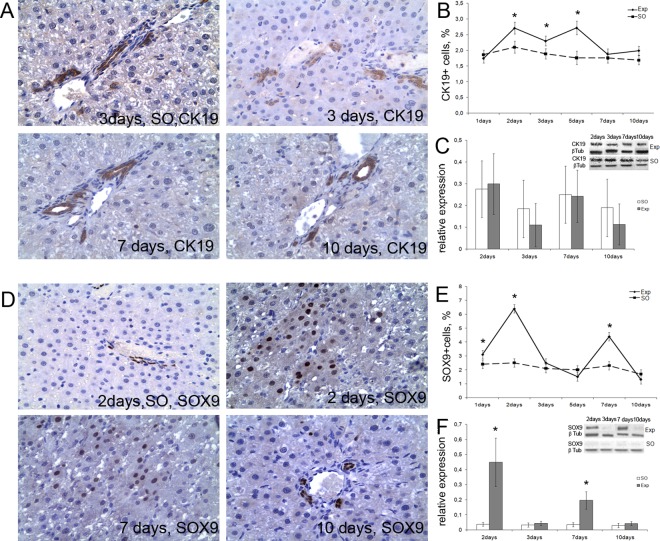
Expression of CK19 and Sox9 proteins in the residual liver tissue after subtotal hepatectomy. (**А**) Relative CK19^+^ cell counts for sham operated liver in comparison with residual livers at different stages of regeneration measured by time elapsed after the surgery. (**B**) The diagram shows CK19^+^ index changing in the course of regeneration. (**С**) Evaluation of CK19 protein expression by western blot followed by quantitative densitometry. (**D**) Sox9^+^ cells in the sham operated liver tissue and in the residual livers at different stages of regeneration. Whereas Sox9 protein is found exclusively in the nuclei of cholangiocytes in the sham operated rat livers, it is expressed in the nuclei of hepatocytes after 80% subtotal resection. (**E**) The diagram shows Sox9^+^ index changing in the course of regeneration (**F**). Evaluation of Sox9 protein expression by western blot followed by quantitative densitometry. Exp–operated rats, SO—sham operated rats. The data are represented as mean values ± SEM with asterisks indicating statistical significance of differences (as compared to the control; *p*<0.05).

CK19 antigen was continuously detected in biliary epithelium lining ducts of various diameters (Fig 2A). Although the counts of CK19^+^ cells substantially increased at 2–5 days and returned to their initial values by 7–10 days after the surgery (Fig 2B), these dynamics were not paralleled by significant changes in amount of CK19 protein, as revealed by western blot analysis (Fig 2C).

Sox9 antigen was restricted to cholangiocyte nuclei in the intact liver (Fig 2D); individual Sox9^+^ hepatocytes started to appear by 24 h after the surgery. Subsequently, their numbers peaked twice, at 2 and 7 days after the surgery (Fig 2E). Notably, the western blot staining for Sox9 revealed the same peaks, at 2 and 7 days after the surgery (Fig 2F).

### Gene expression analysis

**Fig 3:**
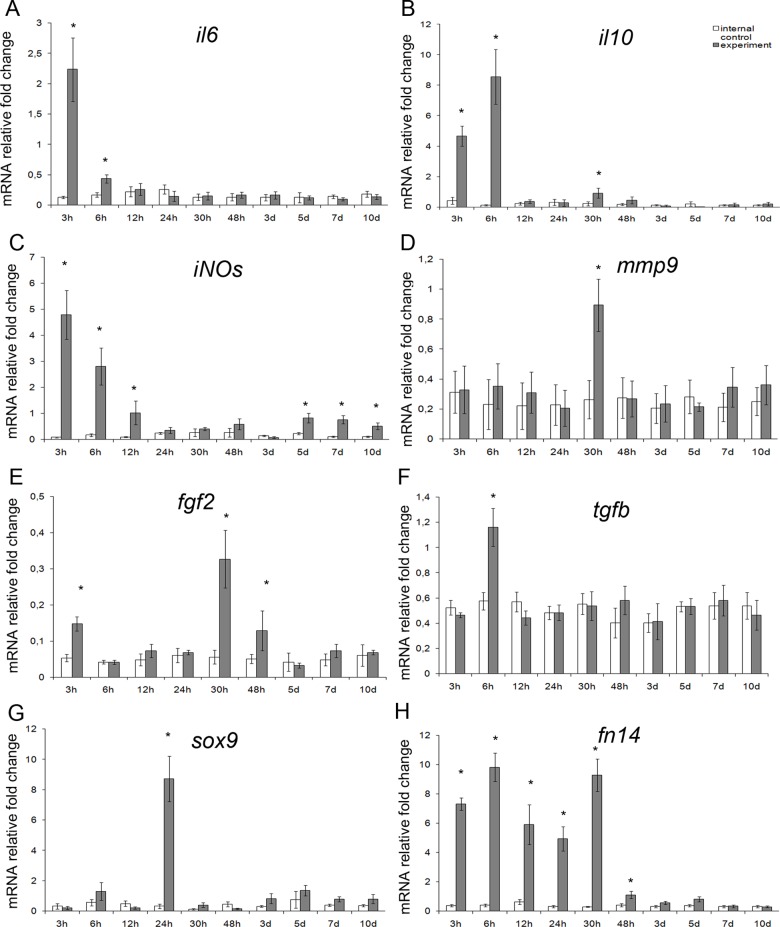
A set of earlier markers with expression upregulated in the residual liver tissue at 5 to 10 days after subtotal hepatectomy. Expression levels for *il6*, *il10*, *iNOs*, *mmp9*, *fgf2*, *tgfb*, *sox9*, and *fn14* (respectively **A**, **B**, **C**, **D**, **E**, **F**, **G**, and **H**) are plotted against time elapsed after the surgery. The data are represented as mean values ± SEM with asterisks indicating statistical significance of differences (as compared to respective controls; *p*<0.05).

**Fig 4:**
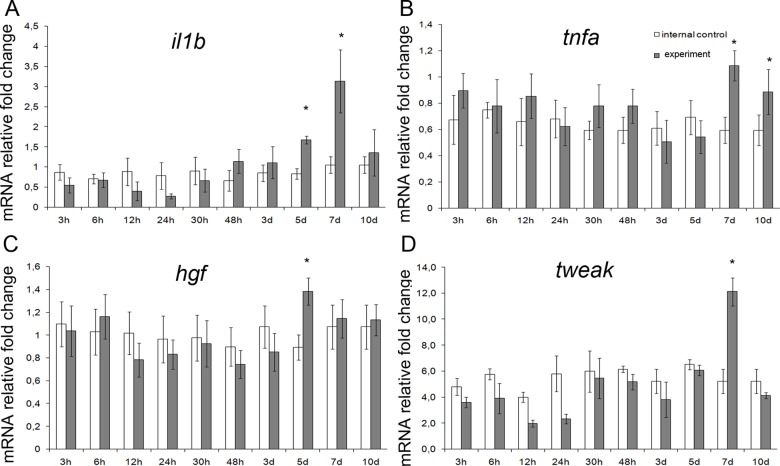
A set of later markers with expression upregulated in the residual liver tissue at 5 to 10 days after subtotal hepatectomy. Expression levels for *il1b*, *tnfa*, *hgf*, and *tweak* (respectively **A**, **B**, **C**, and **D**) are plotted against time elapsed after the surgery. The data are represented as mean values ± SEM with asterisks indicating statistical significance of differences (as compared to respective controls; *p*<0.05).

**Fig 5:**
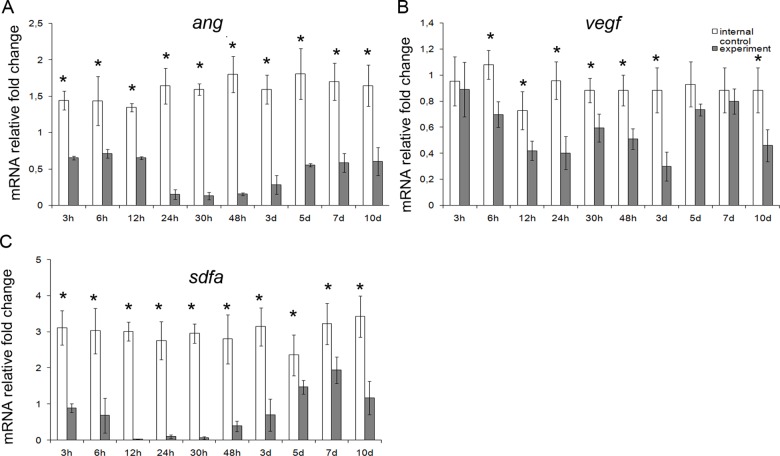
A set of markers with expression downregulated in the residual liver tissue. (**A**) Expression levels for *ang*, *vegf*, and *sdfa* (respectively **A**, **B**, and **C**) are plotted against time elapsed after the. The data are represented as mean values ± SEM with asterisks indicating statistical significance of differences (as compared to respective controls; *p*<0.05).

Upon analysis, the genes split up into three groups. The first group comprised mRNA species over-represented in hepatic tissues during the early period (3 to 48 h after the surgery); these were *il6*, *il10*, *iNOs*, *mmp9*, *fgf2*, *tgfb*, *fn14* and *sox9* (Fig 3). The second group comprised mRNA species over-represented in hepatic tissues during the late period (5 to 10 days after the surgery); these were *il1b*, *tnfα*, *tweak*, *iNOs*, and *hgf* (Fig 4). The third group, opposing the first two, comprised mRNA species significantly under-represented in hepatic tissues for a certain time period during the regeneration; these were *ang*, *vegf*, and *sdfa* (Fig 5).

**Fig 6:**
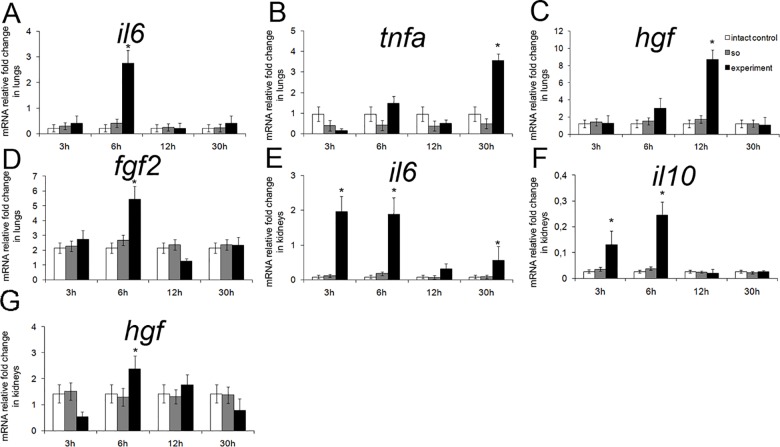
A set of markers with expression upregulated in lungs and kidneys after subtotal hepatectomy. Expression levels for *il16*, *tnfa*, *hgf*, and *fgf2* in lungs (respectively, **A**, **B**, **C**, and **D**) and for *il16*, *il10* and *hgf* in kidneys (respectively **E**, **F**, and **G**) are plotted against time elapsed after the surgery in comparison with sham operated animals. Exp–operated rats, SO—sham operated rats. The data are represented as mean values ± SEM with asterisks indicating statistical significance of differences (as compared to respective controls; *p*<0.05).

Additionally, transcription of some genes was significantly upregulated in lungs and kidneys. For example, increased expression of *il6*, *hgf*, and *fgf2* in the lungs was detected at 6 h after the surgery, and increased expression of *tnfa*—at 30 h after the surgery. Similarly, the *il6*, *il10*, and *hgf* were upregulated in the kidneys at 6 h after the surgery (Fig 6).

## Discussion

Subtotal hepatectomy represents a severe trauma for a rat, hence the high mortality of experimental animals. In surviving animals, residual livers gradually increased in size, reaching the weight of normal liver by 10 days after the surgery. The most rapid growth was recorded between 3 and 7 days after the surgery, while the biochemical indexes of hepatic function approached their normal values by the end of this period. The description is consistent with literary data [6]; generally, the events were similar to those for partial hepatectomy [9].

At the same time, only few mitotic divisions were observed in hepatocytes during the first 36 h after the surgery (Fig 1C). This is consistent with an idea that proliferation of hepatocytes after the 80% subtotal hepatectomy is regulated differently as compared to the partial hepatectomy, so that hepatocytes with mitotic figures in subtotally hepatectomized rats can be observed at about 36 h after the surgery, whereas in partially hepatectomized rats they can be observed already within 12 h, and their numbers peak at 24 h after the surgery; the delay is explained by G0 or G2/M cell cycle arrest [24].

Mitotic index curve for the subtotal hepatectomy-induced hepatocyte proliferation is shown in Fig 1C. It is exhibiting two distinctive maxima at 48–72 h and 7 days after the surgery. The second peak indicates some mechanism for cell synchronization on later stages. Mitotic figures in hepatocytes were still present at 10 days after the surgery (Fig 1C), despite complete regain of the initial hepatic tissue volume. The partial hepatectomy also results in two peaks of hepatocyte proliferation, but these fall within the first two days after the surgery [9]. By contrast, several peaks of hepatocyte proliferation are observed after the partial hepatectomy in mice; collectively, these peaks span both early and late periods of the regeneration (48 and 72 h, and 4 and 5 days, respectively) [25]. Being compared with the results of current study, these data emphasize the existence of species-specific features in the hepatectomy-induced hepatocyte proliferation.

Molecular pathways that control liver regeneration in mammals are studied mainly on the partial hepatectomy model [8,9]. The immediate early gene expression changes are associated with the reaction of liver tissue to metabolic failure associated with the acute injury, and this condition subsequently proceeds into a primary stage of recovery. Accordingly, the increased expression of cytokines that regulate tissue inflammation, yet also the entry of hepatocytes into mitotic cycle, is subsequently joined by the increased expression of growth factors that maintain hepatocyte proliferation [7,8].

For the partial hepatectomy model, increased expression of Hgf, Tgfb, and some other growth factor-encoding genes in hepatic tissue is maintained for no longer than 48 h after the surgery [9]. Time limits obtained for 80% subtotal hepatectomy model in this study are not that definite: increased expression is maintained for 3 days after the surgery [6], but may last longer, for up to 14 days [10,11,12].

The early and the late phases of upregulated gene expression, described in this report, span the intervals from 3 to 48 h and from 5 to 10 days after the surgery, respectively. These phases may be associated with the two waves of hepatocyte proliferation. Obvious differences in the lists of upregulated genes may be attributed to several factors. First, the initial stage of liver regeneration is always marked by an increase in expression of so-called "acute phase genes" (which include Il1b, Il6, and Il10) and some functionally related genes (e.g. Mmp9) [8,9]. The reported data is consistent with this knowledge; Fig 3A and 3B give an example of immediate early genes Il6 and Il10, substantially upregulated already at 3–6 h after the surgery. Second, the expression of some genes encoding growth factors is influenced by availability of these factors from deposits on demand (e.g. by release from the extracellular matrix upon the organ damage). A typical example is presented by hepatocyte growth factor (HGF), the major hepatocyte-specific mitogen in liver regeneration, encoded by Hgf gene [9]. Transcription of this gene starts to rise only as a consequence of depletion of the factor, but if the stocks are low initially, it can be readily upregulated at the early stages. This may also be the case for Tgfb gene that encodes transforming growth factor beta (TGFβ), which counteracts excessive stimulation of reparative processes by a weak inhibitory action, or Fgf2 gene that encodes basic fibroblast growth factor (bFGF), which acts as a weak hepatocyte- and endotheliocyte-specific mitogen [1,9]. Third, transcription of some genes in the regenerating hepatic tissue may depend on regulation by hormones or factors supplied from other organs. For example, HGF is intensively produced in lungs, kidneys, and spleen shortly after the partial hepatectomy [26,27]. Accordingly, we detected the increased expression of several genes (including Il6, Il10, Tnf, Hgf, and Fgf2) in lungs and kidneys at the early stages (Fig 6). Although the increased production of these factors in lungs and kidneys is possibly caused by major metabolic disturbances (arising from the liver failure), it may influence their production in the regenerating hepatic tissue. The impact of extra-hepatic pool of growth factors and cytokines apparently depends on their amounts and also on whether they act locally or are secreted into the bloodstream.

In addition, since some of the genes with increased expression (Il1b, Il6, Il10, Nos2 (*iNOs*), Tnfa, and Tnfsf12 (*tweak*); see Figs 3 and 4) are known to be expressed in macrophages, the results are consistent with the coordinating role in liver regeneration assigned to the resident populations of macrophages, predominantly the Kupffer cells [8,9].

According to published data, tumor necrosis factor alpha (TNFα) in conjunction with interleukin 6 (IL6) form a regulatory system that forces the hepatocytes into mitotic cycle by inducing the acute phase gene expression [7,8]. TNFα and IL6 are also shown to stimulate the synthesis of inducible nitric oxide synthase (the iNOS protein encoded by Nos2 gene), which counteracts their influence by a negative feedback [28]. Furthermore, IL6 may act as an inducer of HGF synthesis, exert anti-inflammatory action in conjunction with IL10, stimulate proliferation of epithelial cells, and suppress the apoptosis [29].

The *Fgf2*-encoded bFGF is characterized by wide spectrum of targets and modes of action in stimulating cell growth and differentiation (including specific tasks e.g. angiogenesis). The cross-talk between cytokines and bFGF is being extensively studied. It is shown that anti-inflammatory М2 macrophages are capable of inducing *Fgf2* expression in tissues [30], whereas the pro-inflammatory IL1b can stimulate secretion of bFGF by endotheliocytes [31]. Although the roles of IL1b and IL10 in liver regeneration are not yet fully understood, it is shown that IL1b and IL10 synergistically inhibit hepatocyte proliferation, despite their basic antagonism to inflammatory processes. Thus, IL1b and IL10 in conjunction with TGFβ promote completion of reparative processes in the liver [32].

By contrast, genes Vegfa (*vegf*), Cxcl12 (*sdfa*), and Ang2 (*ang*) were expressed in the regenerating liver at lower levels than in the intact liver (Fig 5). Of proteins encoded by these genes, VEGF-A and angiogenin stimulate endotheliocyte proliferation in the regenerating liver [33], whereas SDF1a causes migration of multipotent cells from bone marrow to the regenerating liver [34]. How to reconcile these functions of the proteins with the decreased expression of the corresponding genes is as yet unclear, but perhaps these decreases are specific to the subtotal hepatectomy, and it is possible that they occur with participation of microRNA [35].

Currently, much of the attention is drawn to alternative mechanisms of liver regeneration that are not directly related to the proliferation of hepatocytes. Some authors suggest the presence of a particular type of liver cells, representing a common progenitor of hepatocytes and cholangiocytes. Perhaps these are the oval cells, which are identified in the hepatic parenchyma of rodents, where they constitute a part of the intralobular bile ductules (canals of Hering). Phenotypically, the oval cells are close to cholangiocytes since they express CK19 and several other cytokeratines, and also the transcription factor Sox9 [13,16,36]. Some authors believe that all cholangiocytes are facultative HPC, which can be induced by damage to the biliary epithelium. That is, upon hepatic trauma some cholangiocytes may undergo reprogramming and give rise to populations of the oval cells [37,38]. According to Bird *et al*. [16], the reprogramming is accompanied by an increase in numbers of cells expressing cholangiocyte-specific molecules (e.g. CK19), and, reciprocally, such an increase should indicate the HPC activation. As a consequence, the cells with dual molecular phenotype i.e. expressing markers of both cholangiocytes (CK19, panCK) and hepatocytes (NHF4, albumin) start to appear within the parenchyma; noteworthy, these cells can be seen not only in the ducts but also within the lobules [37,16].

However, the plasticity of cholangiocytes may be considered secondary, since in certain circumstances cholangiocytes derive from hepatocytes as a result of reprogramming of the latter [37,39]. This sort of reprogramming is typical for experimental models of toxic injury to the liver or during the obstructive cholestasis in the bile duct-ligated animals. The order of events is as follows: at first there appears a substantial number of hepatocytes expressing Sox9, and subsequently the number of cells with the dual hepatocyte-cholangiocyte phenotype increases [37,39]. It is assumed that the leading role in the regulation of hepatocyte reprogramming is played by Notch-signaling [37,39].

Thus, the choice of cell sources used for the repair largely depends on severity and nature of the damage. In cases of blocked hepatocyte proliferation the role of their precursors goes to cholangiocytes or their derivatives, while in cases of extensive damage to biliary epithelium the reprogrammed hepatocytes serve as precursors for cholangiocytes.

In the current study, CK19 protein expression was observed in the biliary epithelium of the ducts but never beyond it–even at the increases in total proportion of CK19^+^ cells (Fig 2A). At the same time, the CK19 protein content of liver tissue revealed by western blot analysis did not significantly rise from the baseline during the entire period of regeneration (Fig 2C). According to [37,16], this may indicate predominance of the biliary epithelium hyperplasia over the processes of HPC activation.

A characteristic histological feature of regeneration described in the current study was the appearance of a large number of cells with the morphology of hepatocytes, which were, however, Sox9-positive (as revealed by immunostaining, Fig 2D). This may indicate the reprogramming of hepatocytes for the subsequent differentiation to cholangiocytes. Notably, the waves of sharp increase in numbers of Sox9^+^ cells were followed by the periods of confinement of Sox9 protein to the biliary epithelium (see Fig 2), and no accumulation of the cells with intermediate or dual phenotypes was observed in the lobular parenchyma. This is consistent with the CK19 immunostaining data: the majority of CK19^+^ cells had the typical cholangiocyte morphology and was located in the bile ducts.

Therefore, neither the activation of resident progenitor cells nor the reprogramming of hepatocytes for subsequent differentiation to cholangiocytes can be excluded for the subtotal hepatectomy-induced regeneration. The former is indicated by the appearance of Sox9^+^ hepatocytes, and the latter is evidenced by the increased numbers of CK19^+^ cells on the background of increased Tnfrsf12a and Tnfsf12 gene expression. But, whatever might be interrelation of these processes, they apparently play some auxillary roles in the subtotal hepatectomy-induced liver regeneration, whereas the proliferation of hepatocytes serves the main route for the organ recovery.

Notably, the peaks of hepatocyte mitotic index coincided with the maxima of Sox9^+^ hepatocyte counts (Figs 1C, 2D and 2E). Sox9 transcription factor plays an important role in mammalian histogenesis. In the postnatal ontogeny it is specifically expressed by some populations of highly-proliferative undifferentiated cells such as spermatogonia or epithelial cells at the bottom of intestinal crypts [13]. It is known that hepatocytes, prior to the entry into mitotic cycle, dedifferentiate to a certain point when they start to express a set of hepatoblast markers including α-fetoprotein [1]. The emergence of Sox9 protein in hepatocytes may reflect their “intention” to proliferate–hence the coincidence.

The TWEAK/Fn14 pathway is implicated in coordinating cholangiocyte proliferation, although some studies also indicate participation of the TWEAK/Fn14 signaling in activation of resident HPC [16]. Upregulated transcription of genes encoding Fn14 (from 30 min to 48 h after the surgery) and TWEAK (as peaks at 7 days after the surgery) was observed in this study (Fig 3H). In the absence of distinct evidence of HPC activation, modulation of TWEAK/Fn14-signaling may reflect its participation in the hepatocyte and cholangiocyte proliferation management rather than in the activation of undifferentiated progenitors [40].

## Conclusions

The study revealed several characteristic features of the subtotal hepatectomy-induced liver regeneration in rats, which was subdivided into periods due to the presence of two peaks of hepatocyte proliferation. The first of them was observed in the early period, at 48 to 72 h after the surgery, and the second peak was observed at 7 days after the surgery. Two phases of upregulated gene transcription turned out to be associated with these peaks. The first phase was marked by upregulated expression of Mmp9, Il6, Il10, Fgf2, Tnfrsf12a (Fn14), and Tgfb; the second phase was marked by upregulated expression of Hgf, Tnfa, Tnfsf12 (TWEAK), and Il1b.

Recognition and selective analysis of adult stem cells in the liver admittedly represent a complex methodological problem due to the lack of specific markers. Searches for such markers, including the ‘omic’ approaches, were undertaken repeatedly but unsuccessfully. According to the established concept, the adult stem cells of the liver, the HPCs, express specific markers of both hepatocytes and cholangiocytes.

In the mammalian liver, cholangiocytes are much inferior to hepatocytes by number, so that changes in representation of cholangiocytes and their markers can be more clearly assessed against the bulk of hepatocytes. Therefore, it is appropriate to trace the presence of HPCs by using the cholangiocyte-specific markers–which, of course, is only a convenient assumption, because, in fact, it reveals a mixed set of HPCs and cholangiocytes, unless a thorough morphological evaluation is available (the completeness of such evaluations is always more or less conventional). Thus, a sharp increase in the number of cells expressing markers of cholangiocytes in mammalian liver during reparative processes or pathological conditions is commonly regarded as the activation of alternative pathways of liver regeneration [13,16].

Adhering to this concept, we have attempted to trace the course of the regeneration by monitoring the expression of cholangiocyte markers CK19 and Sox9. During the observation period, the changes in CK19 expression were minimal, and the cells were invariably incorporated in the bile ducts, while the Sox9 protein expression was additionally detected in hepatocytes (strictly, in the cells morphologically identical to hepatocytes and located inside the plates) at some stages. The proportion of CK19+ cells at these stages remained constantly low; therefore, we assume that the expression of Sox9 in hepatocytes is more related with their preparation to proliferate than dedifferentiation.

Comprehension of the obtained data allows us to conclude that the activation of alternative pathways of liver regeneration after the subtotal resection is minor, and the organ mass is restored by mature hepatocyte proliferation driven by its classical regulators Il6, Hgf, Tgf-β etc. Subtotal character of the resection has a significant impact on the dynamics of the mitotic reproduction of hepatocytes, which is reflected in the reversible mitotic cycle arrest and the delayed emergence of mitotically active hepatocytes. These specific effects are plausibly supported by compensatory molecular signaling networks that regulate hepatocyte proliferation. One of the possible mechanisms in this case is the TWEAK/Fn14-signaling pathway, activation of gene expression for which has been observed in the experiment.

Despite the fact that hepatocyte proliferation turned to be a leading process in liver regeneration after the subtotal 80% hepatectomy in rats, the data did not allow to exclude the HPCs participation. According to the literature, the HPCs distinctly participate in the regeneration even after the partial 70% liver resection [13,36]; it is that their contribution to the restoration of the organ mass, similarly to the case of partial resection, is not a major one.

## Supporting Information

S1 Fig A. Mortality in groups. The diagram represents the absolute numbers of operated animals, as well as the absolute numbers of surviving and dead animals for all groups. Some of the animals died over the first two days after the surgery giving total mortality of approx. 50%. The data are represented as absolute numbers of animals, h–hours, d—days. B. Distribution of the spontaneous deaths in time after the surgery. The plot represents relative mortality (i.e. the number of deaths that occurred in a given interval divided by the total number of spontaneous deaths) for sequential intervals between time points after the surgery. Among the dead rats most of the animals died within 48 hours after the surgery. The data are represented as percentage of dead animals, h–hours, d—days.
(TIF)

S1 File Ethics statement. (DOC)

## References

[1] Michalopoulos GK, DeFrances MC. Liver regeneration. Science. 1997;276(5309): 60–66. 9082986 10.1126/science.276.5309.60

[2] http://hilltoplabs.com/public/subtotalhepatectomy.html. Accessed 05 February 2016.

[3] Mitchell C, Nivison M, Jackson LF, Fox R, Lee DC, Campbell JS, Fausto N. Heparin-binding epidermal growth factor-like growth factor links hepatocyte priming with cell cycle progression during liver regeneration J Biol Chem. 2005;280(4): 2562–2568. 15536070 10.1074/jbc.M412372200

[4] Ninomiya M., Shirabe K, Terashi T, Ijichi H, Yonemura Y, Harada N, et al Deceleration of regenerative response improves the outcome of rat with massive hepatectomy. Am J Transplant. 2010;10(7): 1580–1587. 10.1111/j.1600-6143.2010.03150.x 20642684

[5] Eshkenazy R, Dreznik Y, Lahat E, Zakai BB, Zendel A, Ariche A. Small for size liver remnant following resection: prevention and management. Hepatobiliary Surg Nutr. 2014;3: 303–312. 10.3978/j.issn.2304-3881.2014.09.08 25392842 PMC4207847

[6] Sowa JP, Best J, Benko T, Bockhorn M, Gu Y, Niehues EM, et al Extent of liver resection modulates the activation of transcription factors and the production of cytokines involved in liver regeneration. World J Gastroenterol. 2008;14(46): 7093–7100. 19084916 10.3748/wjg.14.7093 PMC2776839

[7] Fausto N. Liver Regeneration. J Hepatol. 2000;32(1 Suppl): 19–31. 10728791 10.1016/s0168-8278(00)80412-2

[8] Fausto N, Campbell JS, Riehle KJ. Liver Regeneration. Hepatology. 2006;43(2 Suppl 1): S45–53. 16447274 10.1002/hep.20969

[9] Michalopoulos GK. Advances in liver regeneration. Expert Rev Gastroenterol Hepatol. 2014;8(8): 897–907. 10.1586/17474124.2014.934358 24964729

[10] Panis Y, Lomri N, Emond JC. Early gene expression associated with regeneration is intact after massive hepatectomy in rats. J Surg Res. 1998;79(2): 103–108. 9758723 10.1006/jsre.1998.5400

[11] Masson S, Daveau M, Hiron M, Lyoumi S, Lebreton JP, Ténière P, Scotté M. Differential regenerative response and expression of growth factors following hepatectomy of variable extent in rats. Liver. 1999;19(4): 312–317. 10459630 10.1111/j.1478-3231.1999.tb00055.x

[12] Scotté M, Masson S, Lyoumi S, Hiron M, Ténière P, Lebreton JP, Daveau M. Cytokine gene expression in liver following minor or major hepatectomy in rat. Cytokine. 1997;9(11): 859–867. 9367547 10.1006/cyto.1997.0273

[13] Furuyama K, Kawaguchi Y, Akiyama H, Horiguchi M, Kodama S, Kuhara T, et al Continuous cell supply from a Sox9-expressing progenitor zone in adult liver, exocrine pancreas and intestine. Nat Genet. 2011;43(1): 34–41. 10.1038/ng.722 21113154

[14] Michalopoulos GK. Liver Regeneration. J Cell Physiol. 2007; 213(2): 286–300. 10.1002/jcp.21172 17559071 PMC2701258

[15] Han S, Yoon K, Lee K, Kim K, Jang H, Lee NK, et al TNF-related weak inducer of apoptosis receptor, a TNF receptor superfamily member, activates NF-kappa B through TNF receptor-associated factors. Biochem Biophys Res Commun. 2003;305(4): 789–796. 12767899 10.1016/s0006-291x(03)00852-0

[16] Bird TG, Lu WY, Boulter L, Gordon-Keylock S, Ridgway RA, Williams MJ, et al Bone marrow injection stimulates hepatic ductular reactions in the absence of injury via macrophage-mediated TWEAK signaling. Proc Natl Acad Sci U S A. 2013;110(16): 6542–6547. 10.1073/pnas.1302168110 23576749 PMC3631632

[17] Tirnitz-Parker JE, Viebahn CS, Jakubowski A, Klopcic BR, Olynyk JK, Yeoh GC, Knight B. Tumor necrosis factor-like weak inducer of apoptosis is a mitogen for liver progenitor cells. Hepatology. 2010; 52: 291–302. 10.1002/hep.23663 20578156

[18] Boulter L, Lu WY, Forbes SJ. Differentiation of progenitors in the liver: a matter of local choice. J Clin Invest. 2013;123: 1867–1873. 10.1172/JCI66026 23635784 PMC3635730

[19] Jakubowski A, Ambrose C, Parr M, Lincecum JM, Wang MZ, Zheng TS, et al TWEAK induces liver progenitor cell proliferation. J Clin Invest. 2005;115: 2330–2340. 16110324 10.1172/JCI23486 PMC1187931

[20] http://web.jhu.edu/animalcare/policies/ether.html. Accessed 05 February 2016.

[21] Li J, Campbell JS, Mitchell C, McMahan RS, Yu X, JK Riehle, Bumgarner RE, and Fausto N Relationships between Deficits in Tissue Mass and Transcriptional Programs after Partial Hepatectomy in Mice The American Journal of Pathology, Vol. 175, No. 3, 9 2009; 947–957 10.2353/ajpath.2009.090043 19700759 PMC2731115

[22] Pfaffl MW. A new mathematical model for relative quantification in real-time RT-PCR. Nucleic Acids Res. 2001;29(9): e45 11328886 10.1093/nar/29.9.e45 PMC55695

[23] Vandesompele J, De Preter K, Pattyn F, Poppe B, Van Roy N, De Paepe A, Speleman F. Accurate normalization of real-time quantitative RT-PCR data by geometric averaging of multiple internal control genes. Genome Biol. 2002;3(7): RESEARCH0034 12184808 10.1186/gb-2002-3-7-research0034 PMC126239

[24] Weinbren K, Taghizadeh A. The mitotic response after subtotal hepatectomy in the rat. Br J Exp Pathol. 1965;46(4): 413–417. 5825776 PMC2094607

[25] Zou Y, Bao Q, Kumar S, Hu M, Wang GY, Dai G. Four waves of hepatocyte proliferation linked with three waves of hepatic fat accumulation during partial hepatectomy-induced liver regeneration. PLoS ONE. 2012;7(2): e30675 10.1371/journal.pone.0030675 22319576 PMC3272022

[26] Yanagita K., Nagaike M., Ishibashi H. Lung may have an endocrine function producing hepatocyte growth factor in response to injury of distal organs. Biochem Biophys Res Commun. 1992;182(2): 802–809. 1531175 10.1016/0006-291x(92)91803-x

[27] Kono S, Nagaike M, Matsumoto K, Nakamura T. Marked induction of hepatocyte growth factor mRNA in intact kidney and spleen in response to injury of distant organs. Biochem Biophys Res Commun. 1992; 186(2): 991–998. 1379811 10.1016/0006-291x(92)90844-b

[28] Rai RM, Lee FYJ, Rosen A, Yang SQ, Lin HZ, Koteish A, et al Impaired liver regeneration in inducible nitric oxide synthase deficient mice. Proc Nat Acad Sci U S A. 1998;95: 13829–13834. 10.1073/pnas.95.23.13829 PMC24912 9811886

[29] Coudriet GM, He J, Trucco M, Mars WM, Piganelli JD. Hepatocyte growth factor modulates interleukin-6 production in bone marrow derived macrophages: implications for inflammatory mediated diseases. PLoS ONE. 2010;5(11): e15384 10.1371/journal.pone.0015384 21072211 PMC2970559

[30] Jetten N, Verbruggen S, Gijbels MJ, Post MJ, De Winther MP, Donners MM. Anti-inflammatory M2, but not pro-inflammatory M1 macrophages promote angiogenesis in vivo. Angiogenesis. 2014;17(1): 109–118. 10.1007/s10456-013-9381-6 24013945

[31] Presta M, Dell'Era P, Mitola S, Moroni E, Ronca R, Rusnati M. Fibroblast growth factor/fibroblast growth factor receptor system in angiogenesis. Cytokine Growth Factor Rev. 2005;16(2): 159–178. 15863032 10.1016/j.cytogfr.2005.01.004

[32] Yin S, Wang H, Park O, Wei W, Shen J, Gao B. Enhanced liver regeneration in IL-10-deficient mice after partial hepatectomy via stimulating inflammatory response and activating hepatocyte STAT3. Am J Pathol. 2011;178(4): 1614–1621. 10.1016/j.ajpath.2011.01.001 21435447 PMC3078469

[33] Leve LD. Liver sinusoidal endothelial cells and liver regeneration J Clin Invest. 2013;123(5): 1861–1866. 10.1172/JCI66025 23635783 PMC3635729

[34] Dalgetty DM, Medine CN, Iredale JP, Hay DC. Progress and future challenges in stem cell-derived liver technologies Am J Physiol Gastrointest Liver Physiol. 2009;297: G241–G248. 10.1152/ajpgi.00138.2009 19520740

[35] Chen Y, Verfaillie CM. MicroRNAs: the fine modulators of liver development and function. Liver Int. 2014;34(7): 976–990. 10.1111/liv.12496 24517588

[36] Malato Y, Naqvi S, Schürmann N, Ng R, Wang B, Zape J, et al Fate tracing of mature hepatocytes in mouse liver homeostasis and regeneration. J Clin Invest. 2011;121(12): 4850–4860. 10.1172/JCI59261 22105172 PMC3226005

[37] Yanger K, Zong Y, Maggs LR, Shapira SN, Maddipati R, Aiello NM, et al Robust cellular reprogramming occurs spontaneously during liver regeneration. Genes Dev. 2013;27(7): 719–724. 10.1101/gad.207803.112 23520387 PMC3639413

[38] Rodrigo-Torres D, Affò S, Coll M, Morales-Ibanez O, Millán C, Blaya D, et al The biliary epithelium gives rise to liver progenitor cells. Hepatology. 2014;60(4): 1367–1377. 10.1002/hep.27078 24700364 PMC4410184

[39] Yimlamai D, Christodoulou C, Galli GG, Yanger K, Pepe-Mooney B, Gurung B, et al Hippo pathway activity influences liver cell fate. Cell. 2014;157(6): 1324–1338. 10.1016/j.cell.2014.03.060 24906150 PMC4136468

[40] Karaca G, Swiderska-Syn M, Xie G, Syn WK, Krüger L, Machado MV, et al TWEAK/Fn14 signaling is required for liver regeneration after partial hepatectomy in mice. PLoS ONE. 2014;9(1): e83987 10.1371/journal.pone.0083987 24416188 PMC3886973

