# Supplementary figures and images for: Molecular Survey of Cell Source Usage during Subtotal Hepatectomy-Induced Liver Regeneration in Rats
Source: PLoS One. 2016 Sep 15;11(9):e0162613. doi: 10.1371/journal.pone.0162613 (PMC5025203; doi:10.1371/journal.pone.0162613)

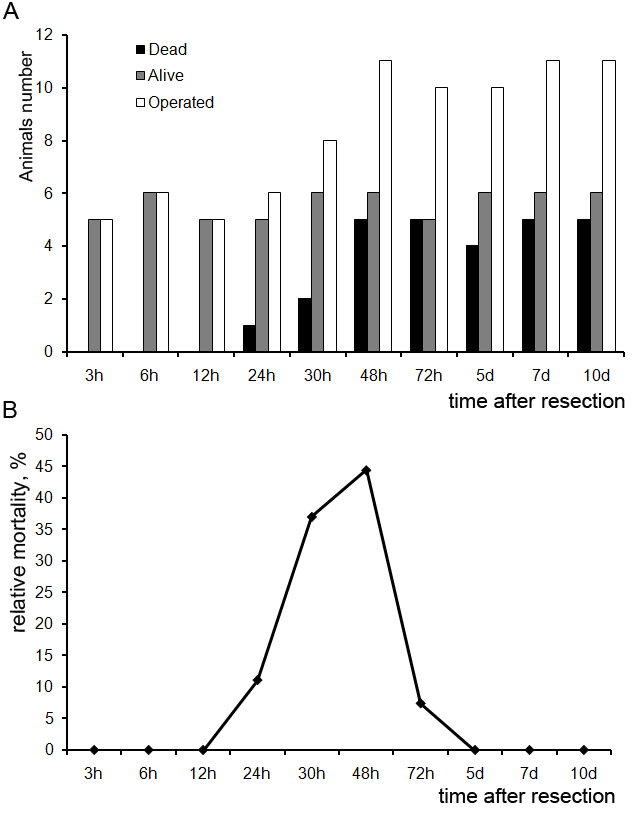

Supplement: S1 Fig — A. Mortality in groups. The diagram represents the absolute numbers of operated animals, as well as the absolute numbers of surviving and dead animals for all groups. Some of the animals died over the first two days after the surgery giving total mortality of approx. 50%. The data are represented as absolute numbers of animals, h–hours, d—days. B. Distribution of the spontaneous deaths in time after the surgery. The plot represents relative mortality (i.e. the number of deaths that occurred in a given interval divided by the total number of spontaneous deaths) for sequential intervals between time points after the surgery. Among the dead rats most of the animals died within 48 hours after the surgery. The data are represented as percentage of dead animals, h–hours, d—days. (TIF) [file pone.0162613.s001.tif]
