# Supplementary material for: Molecular Survey of Cell Source Usage during Subtotal Hepatectomy-Induced Liver Regeneration in Rats
Source: PLoS One. 2016 Sep 15;11(9):e0162613. doi: 10.1371/journal.pone.0162613 (PMC5025203; doi:10.1371/journal.pone.0162613)
Supplement: S1 File — (DOC) [file pone.0162613.s002.doc]

**Ethics statement**

Handling of the animals was carried out according to the standards of laboratory practice (National Guidelines No. 267 by Ministry of Healthcare of the Russian Federation, June 1, 2003). The study was approved by Ethical Board at the Institute of Human Morphology (Protocol No. 5, March 12, 2013), and all efforts were made to minimize the suffering. The outbred male Sprague-Dawley rats of 300-400 g total body mass were used in the experiments. Two animals per cage were housed in a temperature-regulated room with a 12:12 h light-dark cycle, and unlimited access to food and water. The animals were drawn from the experiment in CO_2_-chamber at 3 h, 6 h, 12 h, 24 h, 30 h, 48 h, 72 h, 5 days, 7 days, or 10 days after the surgery. The surgery was performed between 08:00 am and 11:00 pm to minimize potential variability in the progression of liver regeneration associated with surgery time and the circadian clock. The borderline condition produced by subtotal 80% hepatectomy in a rat is described as an acute liver failure; it is externally manifested as reduced overall mobility of the animal combined with piloerection. The condition is resolved in the course of 48 h by either spontaneous death of the animal or switch to the rapid recovery, and no means for apriori distinction between the survivors and non-survivors have been reported. In connection with this layout, the animal health was inspected 4 times a day during the first 48 h after the surgery, and subsequently 2 times a day until the sacrifice.

The operated animals, two per cage, were housed for recovery in a temperature-regulated room with a 12:12 h light-dark cycle and unlimited access to food and water. The animal health was inspected 4 times a day during the first 48 h after the surgery, and subsequently 2 times a day until the sacrifice. Meloxicam (1.0 mg/kg/day) was repeatedly injected into the neck fat pad of the animals as postoperative analgesia for two days after the surgery; additionally, gentamicin (3.0 mg/kg/day) was injected subcutaneously as an antibiotic on the first day after the surgery.

S1 Fig Spontaneous mortality among the operated animals.

А. Mortality in groups. The diagram represents the absolute numbers of operated animals, as well as the absolute numbers of surviving and dead animals for all groups. Some of the animals died over the first two days after the surgery giving total mortality of approx. 50%. The data are represented as absolute numbers of animals, h – hours, d - days

B. Distribution of the spontaneous deaths in time after the surgery. The plot represents relative mortality (i.e. the number of deaths that occurred in a given interval divided by the total number of spontaneous deaths) for sequential intervals between time points after the surgery. Among the dead rats most of the animals died within 48 hours after the surgery. The data are represented as percentage of dead animals, h – hours, d - days
